# Food and Exercise

**Published:** 1856-07

**Authors:** 


### FOOD AND EXERCISE.

In our book on *Bronchitis and Kindred Diseases*, page seventy-seven, eighth Edition, we have selected an "incident" which we repeat here, as suggestive of important deductions in reference to health. The point to which we wish to direct attention at this time is, that in cases where exercise cannot be taken, comparative health may be enjoyed for a considerable period, amounting to months and years, if during that time, the person would eat in moderation, of the plainest and simplest food, allied almost to the "Bread and Water" considered to be "prisoner's fare" of past ages. It will be seen that during the long imprisonment, nothing is intimated to cause us to believe that the unfortunate prisoner was unhealthy, but the reverse.

COUNT CONFALIONERI

wrote from the great jail of *Vienna* as follows:—

"I am an old man now, yet by fifteen years, my soul is younger than my body: fifteen years I existed, for I did not live. It was not life in the self-same dungeon, ten feet square. During six years I had a companion; nine years I was alone. I never could rightly distinguish the face of him who shared my captivity in the eternal twilight of our cell.

"The first year we talked incessantly together. We related our past lives, our joys forever gone, over and over again.

"The next year we communicated to each other our ideas on all subjects.

"The third year we had no ideas to communicate; we were beginning to lose the power of reflection.

"The fourth, at intervals of a month or so, we would open our lips, to ask each other if it were indeed possible that the world was as gay and bustling as it was when we formed a portion of mankind.

"The fifth year we were silent.

"The sixth, he was taken away, I never knew where, to execution or to liberty. But I was glad when he was gone: even solitude was better than that pale and vacant face. After that, I was alone.

"Only one event broke in upon my nine years' vacancy. One day, it must have been a year or two after my companion left me, my dungeon door was opened, and a voice, I knew not whence, uttered these words: 'By order of his Imperial Majesty, I intimate to you, that one year ago, your wife died.' Then the door was shut. I heard no more. They had but flung this great agony in upon me, and left me alone with it again."

The great practical point is this, The only possible way for a lazy man to live in moderate health, is to live upon nothing but bread and water. We must exercise in proportion to our eating.

